# Effects of functional Impairment on dementia caregiver burden across ethnic groups.

**DOI:** 10.1093/geroni/igaf122.2222

**Published:** 2025-12-31

**Authors:** Miriam Jocelyn Rodriguez, Mariana Stavig, Bailey Gardner, Jordan Hill, Lilian Golzarri-Arroyo, Roger Zoh, Richard Holden, Malaz Boustani

**Affiliations:** Indiana University Bloomington, Bloomington, Indiana, United States; Indiana University Bloomington, Bloomington, Indiana, United States; Indiana University Bloomington, Bloomington, Indiana, United States; Indiana University Bloomington, Bloomington, Indiana, United States; Indiana University Bloomington, Bloomington, Indiana, United States; Indiana University Bloomington, Bloomington, Indiana, United States; Indiana University Bloomington, Bloomington, Indiana, United States; Indiana University, Indianapolis, Indiana, United States

## Abstract

**Introduction:**

Functional impairment among individuals with Alzheimer’s disease and related dementias has been associated with increased behavioral and psychological symptoms of dementia (BPSD) and greater caregiver burden. Previous studies have identified greater functional impairment among African American and Hispanic individuals when compared to White non-Hispanic groups. The aim of this study was to examine associations between functional impairment to caregiver burden and depressive symptoms across racial/ethnic groups.

**Method:**

One-way ANOVAs were conducted to compare scores on the Functional Activities Questionnaire (FAQ) between three racial/ethnic groups: White (n = 121), Black/African American (n = 35) and Hispanic/Latino (n = 11). Linear regression analyses were conducted to examine associations between FAQ scores and care recipient BPSD, caregiver burden and depressive symptoms.

**Results:**

The mean of FAQ scores was M = 23 with an SD = 6.51. No significant differences in FAQ scores were found between groups and no associations between FAQ and caregiver burden or depressive symptoms. Significant associations were identified between FAQ scores and care recipient BPSD for the overall sample (F(1,167)=7.93, p<.01; R2= 0.05). Findings were not significant between these two variables and the race/ethnicity of the caregiver.

**Discussion:**

Our findings are consistent with previous research and identified significant associations between functional impairment and BPSD. However, these associations did not impact caregiver burden or depression in our sample. Our sample had limited variation in FAQ scores. Future studies should examine these associations among a sample with a wider range of functional impairment. Our findings have implications for the development of dementia caregiver interventions to best address caregivers’ needs.

